# Role of the keratin 1 and keratin 10 tails in the pathogenesis of ichthyosis hystrix of Curth Macklin

**DOI:** 10.1371/journal.pone.0195792

**Published:** 2018-04-24

**Authors:** Alessandro Terrinoni, Biagio Didona, Sabrina Caporali, Giovanni Chillemi, Alessandro Lo Surdo, Mauro Paradisi, Margherita Annichiarico-Petruzzelli, Eleonora Candi, Sergio Bernardini, Gerry Melino

**Affiliations:** 1 Department of Experimental Medicine and Biochemical Sciences, University of Rome "Tor Vergata", Rome, Italy; 2 Center of Dermatological Rare Diseases, IDI-IRCCS, Via dei Monti di Creta, Rome Italy; 3 SCAI SuperComputing Applications and Innovation Department, Cineca, Via dei Tizii 6, Rome, Italy; 4 Biochemistry Laboratory, IDI-IRCCS, via Monti di Creta, Rome, Italy; Agency for Science Technology and Research, SINGAPORE

## Abstract

Ichthyosis Hystrix of Curth-Macklin (IH-CM) is a rare manifestation of epidermolytic ichthyosis (EI) that is characterised by generalised spiky or verrucous hyperkeratosis. The disorder is further distinguished by the presence of binucleated cells in the affected skin, whereas epidermolysis and clumping of tonofilaments, as seen in EI, are absent. While IH-CM is associated with mutations in the keratin 1 (*KRT1)* gene, reports to date have indicated that mutations in the KRT1 gene result in an aberrant and truncated protein tail, essentially affecting the function of the V2 domain. Here, we studied a female sporadic patient who was born with diffused erythrodermic hyperkeratosis and who presented at the age of 13 months with an intense and widespread hyperkeratosis with a papillomatous appearance and typical palmoplantar keratoderma. Genetic analysis demonstrated a “*de novo*” mutation in the keratin 10 gene (*KRT10)* consisting of a three-base-pair deletion, resulting in the substitution of amino acids p.Glu445 and p.Ile446 by Asp at the end of the 2B domain of the protein. We performed structural and functional studies showing that this mutation modifies the structure of the paired 2B and V2 K1/10 domains, leading to the disease phenotype. Our results highlight the importance and complexity of the KRT1/10 V2 domain in keratin dimer formation and the potential consequences of its alteration.

## Introduction

Ichthyosis hystrix (IH) includes a clinically and genetically heterogeneous group of skin disorders that are characterised by ichthyosis with striking hyperkeratotic and verrucous dark-brown ridges. This form of ichthyosis was first reported in the 1950s [1] and was reassessed in the 70s [2, 3]. A peculiar example of this genodermatosis is the “*porcupine men*'' described by Penrose [4]. In 2000, Braun-Falco suggested the following three clinical forms: (1) Ichthyosis hystrix Curth-Macklin (IH-CM) type, which is characterised by palmoplantar keratoderma with thick, often spiny scales, and a variable extension of the lesions to other parts of the body; (2) Ichthyosis hystrix Lambert type, which displays a phenotype more similar to the porcupine man described in England by Penrose, with spines covering the entire body but sparing the genitalia, face, palms and soles; and [3] Ichthyosis hystrix Rheydt type, which is clinically characterised by hystrix-like hyperkeratosis accompanied by sensorineural deafness and other symptoms suggestive of a non-keratin disease. Recently, a new clinical-genetic classification of the inherited ichthyoses assigned IH-CM to the group of Keratinopathic ichthyosis (KPI), with the acronym ICM [5].

At the histological level, there is a great similarity between the IH-CM and Ichthyosis hystrix Lambert types since these types both exhibit thick hyperkeratosis, papillomatous proliferation, and cellular vacuolisation. Ultrastructural examination of IH-CM patients generally reveals characteristic bi-nucleated cells with thin to thick intermediated keratin tonofilament aggregates (shells) surrounding the nucleus. A clear zone of cytoplasm is present between the tonofilament shells and the nucleus [6].

IH-CM is inherited as an autosomal dominant trait mapped to chromosome 17 and associated with the following mutations in the *KRT1* gene [6]: c1609_16010delGGinsA and c1556delG [6, 7]. Both mutations result in a frameshift of the V2 domain coding region. The c1609_16010delGGinsA mutation [6] was shown to produce an aberrant and truncated protein tail of 77 residues, which lacks seven out of ten glycine loops. The deletion c1556delG leads to a premature termination codon, resulting in a truncated protein [7].

Keratins, which are the major structural proteins of the epidermis, are divided into the following two groups: type I acidic keratins (K9–K20; 17q12–q21), and type II basic keratins (K1–K8; 12q11–q13). From a structural standpoint, keratins contain a central coiled-coil rod domain with four alpha-helical segments (1A, 1B, 2A, and 2B) separated by three non-helical linker elements (L1, L12, and L2) [8]. The central rod domains are highly conserved among species and among keratins of the same class. Non-helical head and tail domains flank the rod domains, and while there is high variability between these domains, they have in common the presence of a glycine loop. The rod domain starts and ends with two short, highly conserved amino acid sequences, known as the "helix initiation peptide" (HIP) and "helix termination peptide" (HTP), respectively [9–11]. The rod domain of keratins is characterised by the repetition of an amino acid heptad sequence (abcdefg). The structure is stabilised by hydrophobic interactions between positions “a” and “d,” and ionic hydrogen interactions between positions “e” and “g.” The “a” residues are thought to interact with amino acids located in the “d” position of the partner molecule of the heterodimer through hydrophobic interactions that stabilise the two-chain coiled-coil molecules (S1 Fig). In the K1/K10 pair, amino acid substitutions modify the initial coupling of keratin dimers [12, 13], and, generally, substitutions in the rod-domain of keratins generate molecular distortion [9] of the alpha-helical structure, with negative effects on KIF formation and also on the integrity of the epidermal structure [14, 15]. For this reason, mutations leading to skin diseases have been frequently mapped to the HIP and the HTP of the central rod domain, representing hot spots for disease-related mutations [16, 17].

Other reports have also demonstrated the role of the variable V1 [18–20] and V2 domains in skin diseases [6, 7, 21]. Models of keratin dimers show that these dimers interact with paired 1A and 2B hetero-domains [22]. This finding suggests a role for the keratin head and tail domains in epidermal differentiation, likely associated with other structural proteins such as SPR or Loricrin [15, 23, 24], and the generation of IF filaments. Computational structural studies have demonstrated an interaction between the paired 1A and 2B domains of K1/10 and their V2 domains, giving rise to a complex structure that is likely responsible for important physiological characteristics of IF filaments [22].

Mutations within different domains of distinct keratins therefore result in diverse pathological phenotypes. For instance, for hystrix ichthyoses, it has been postulated that mutations in *KRT1* result in a phenotype with the presence of palmoplantar keratoderma, the IH-CM type, while mutations in *KRT10* cause the Lambert type (IH-L), in which palmoplantar keratoderma is absent [25, 26].

Here, we have genetically and structurally analysed the effect of a novel mutation in *KRT10* identified in a patient affected by a sporadic form of hystrix ichthyosis of Curth-Macklin. Our results highlight the importance and complexity of the KRT1/10 V2 domain in keratin dimer formation and the potential consequences of its alteration.

## Materials and methods

### Ethics statement

The study was approved by the ethics committee of Fondazione Luigi Maria Monti, IDI-IRCCS, in relationship to Ricerca Corrente, Line-1 project.

The work described was carried out in accordance with The Code of Ethics of the World Medical Association (Declaration of Helsinki). The individual described in this manuscript provided written informed consent to participate in this study; furthermore, the individual described in this manuscript provided their written informed consent (as outlined in PLOS consent form) to publish these case details.

### Light and electron microscopy

Ethical approval and informed consent, according to the Italian Medical-Ethical requirements, were obtained before the skin biopsies were collected. Biopsy samples from the two patients were processed for light microscopy, and samples were paraffin-embedded and stained using haematoxylin-eosin according to standard methods.

### Immunofluorescence and skin histopathology

After treatment, cells were formalin-fixed at room temperature for 10 to 15 min, permeabilised with 0.5% Triton X-100 in PBS for 10 min, washed three times for 10 min in PBS, and incubated for 1 hr in blocking buffer. The following primary antibodies were used for detection: polyclonal anti-p63 (Y4A3 p3362; Sigma-Aldrich; dilution 1:500), polyclonal anti-K10 (Covance; dilution 1:1,000), polyclonal anti-K14 (LL02, Abcam; 1/1000 dilution), and polyclonal anti-Loricrin (Covance; 1/1000 dilution. Fixed skin was embedded in paraffin, and tissue sections were deparaffinised and stained with H&E for histological analysis. For immunohistochemical analyses, sections were deparaffinised, washed in BioClear (Bio-Optica), and rehydrated in solutions with decreasing concentrations of alcohol and increasing concentrations of water. Antigen retrieval was achieved by microwaving the sections in 0.01 M sodium citrate (pH 6). The following secondary antibodies were used to develop immunoreactivities: Alexa Fluor 488 goat anti-rabbit IgG (H+L) antibody and Alexa Fluor 568 goat anti-mouse IgG (H+L) antibody (both from Invitrogen, Carlsbad, CA). Nuclei were stained with DAPI. Cells and tissue sections were mounted using the Prolong Antifade kit (Invitrogen), and slides were analysed with a confocal laser microscope (NIKON Eclipse Ti). Detection of the signal was performed using NIS elements AR4.00.04 software (Nikon).

### Molecular genetic analysis

Genomic DNA was extracted from the patient’s blood according to standard protocols and used for the amplification of the KRT1 and KRT10 codifying regions [13]. Total RNA was extracted from 3-mm skin biopsies, using the RNeasy mini kit (Qiagen, Crawley, UK). RT reactions were performed using Superscript II Reverse Transcriptase (Invitrogen, USA) and 100 ng of total RNA, according to the manufacturer’s instructions, to confirm the genomic mutation. The PCR products were gel purified with the Qiaex extraction kit (Qiagen; UK) and directly sequenced using the amplification primers and additional internal primers.

### Model generation and simulation protocol

The 3D structure of the native K1-K10 heterodimer (rode domain) was obtained from crystallographic data (PDB id 4ZRY) at a resolution of 3.30 Å [27]. The model for the mutation in which E445 and I446 of K10 are mutated in D (445–446) was obtained through the following steps: 1) backbone superimposition of residues 447–456 with residues 446–455; 2) deletion of the original 446–456 residue coordinates and their substitution with residues 447–456 after checking for the absence of interatomic clashes. Therefore, the alpha helical structure of the starting K10 protein was maintained in the starting structure; 3) mutation of residue E445 in D using the Ucsf-Chimera software [35]. The most probable rotamer was chosen after checking for the absence of interatomic clashes.

The starting model was then refined by geometric optimisation followed by molecular dynamic simulations with the Gromacs (v5.0.4) package [36], using the gromos54a7.ff force field [37]. The starting structure was embedded in a dodecahedron box filled with SPC water molecules [38], which extended up to 12 Å from the solute, and counter ions were added to neutralise the overall charge with the gromacs genion tool. After energy minimisation, the system was slowly relaxed for 5 ns, applying positional restraints (1,000 kJ mol^-1^ nm^-2^) to the protein atoms. Then, an unrestrained MD simulation was initiated and extended for 80 ns with a time step of 2 fs. V-rescale temperature coupling was employed to maintain the temperature at a constant 300 K [39]. The simulation was performed enforcing periodic boundary conditions, and the Particle-Mesh Ewald method was used for the treatment of long-range electrostatic interactions [40].

## Results

### Patient clinical presentation

The patient was born at the seventh month of gestation. At birth, she presented a diffused epidermal erythrodermic hyperkeratosis. No other family member was affected by skin pathologies, and the parents were non-consanguineous.

**Fig 1:**
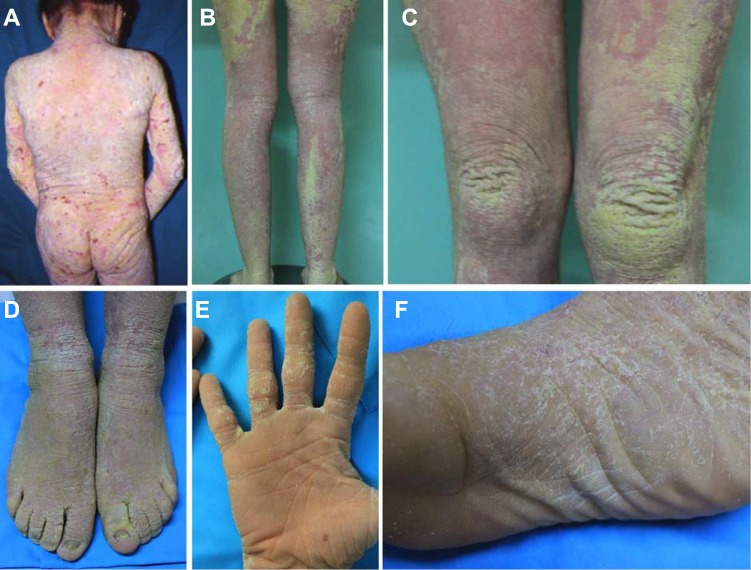
Clinical presentation. (A) Clinical presentation at the age of 13 months, with intense and widespread hyperkeratosis. (B-D) Current clinical presentation, showing a particularly intense hyperkeratosis and erythema along the extensor surfaces of the limbs and on the dorsal region of the hands and feet. (E-F) Palmoplantar hyperkeratosis.

At diagnosis, at the age of 13 months, the patient exhibited an intense and widespread hyperkeratosis with a papillomatous appearance, and only a few areas of the face were spared (Fig 1A). A more recent evaluation of the patient has shown that the clinical disorder is particularly intense along the extensor surfaces of the limbs and on the dorsal region of the hands and feet (Fig 1B–1D), including the palmoplantar areas (Fig 1E and 1F). Clinically, the use of moisturising and mildly keratolytic tonics improved the hyperkeratotic features but did not affect the intensely erythematous areas of the skin. The dermatitis was also accompanied by itching, apparently limited to the affected area. Treatment with emollients did not result in an appreciable improvement of the hyperkeratosis or the erythema.

### Histopathology

**Fig 2:**
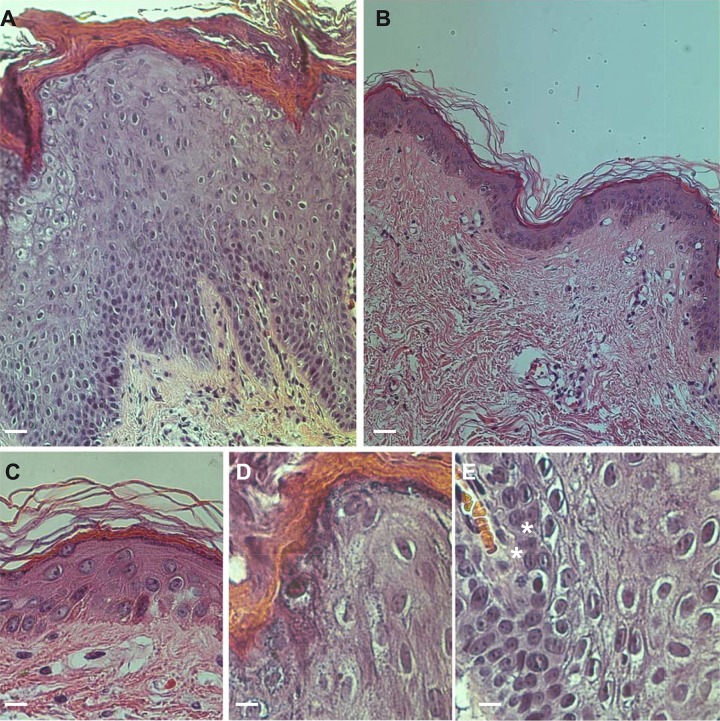
Histopathological analysis. (A) Presence of a strong hyperkeratosis and acanthosis increasing the thickness of the spinous layer with respect to normal controls by over twenty-fold (B) bars = 30 μm. (D-E), significant orthokeratotic hyperkeratosis and presence of parakeratotic cells (C). Intracytoplasmic vacuolisation of the granular layer cells, with features suggestive of epidermolytic hyperkeratosis with a minimal lesion expression (bars = 6 μm).

Histological examination revealed the presence of a strong acanthosis that expanded the spinous layer over twenty-fold, a significant orthokeratotic hyperkeratosis, and, focally, parakeratotic cells (Fig 2C and 2D). Furthermore, there was an intra-cytoplasmic vacuolisation of the granular layer cells, with features characteristic of epidermolytic hyperkeratosis with minimal lesion expression. The dermis exhibited a modest lympho-mononuclear infiltration into the perivascular region.

**Fig 3:**
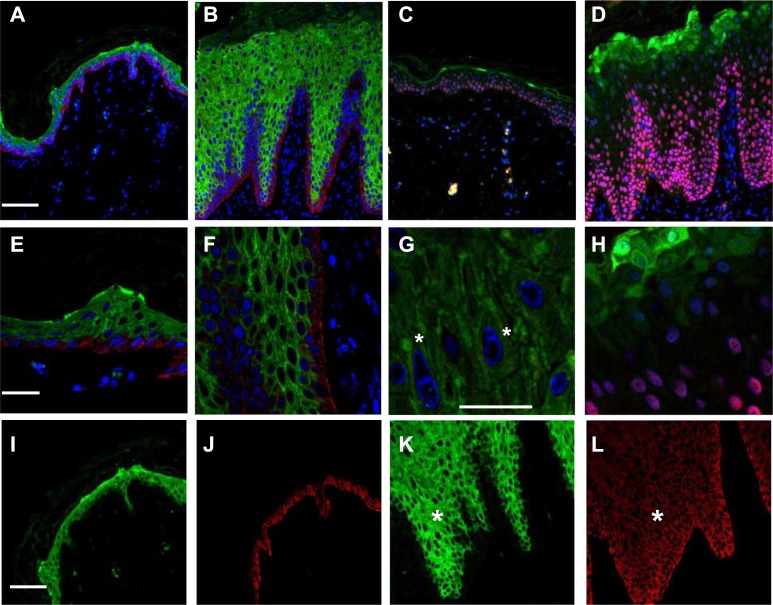
Confocal ultrastructural analysis. (A) Normal and (B) patient skin sections stained with K10 (green) and K14 (red) antibodies revealing abnormal expression of K10. Staining with loricrin (green) and p63 (red) of normal (C) and patient (D) skin sections showing that the basal marker p63 is also expressed in the upper layers; bars = 60 μm. Higher magnification of K 10 and K14 staining showing tonofilament aggregation in the patient skin (F) (E) bars = 20 μm, and a detail of patient K 10 staining showing the binucleated cells in the granular layer (G); bar = 10 μm. Higher magnification of loricrin staining in the patient sample, showing expanded expression and nuclear inclusion (H). Single channel acquisition (K10, green; K14, red) from normal (I-J) and patient skin (K-L) showing a gradient expression of K14 in the upper layers of patient skin, leading to a lighter co-expression of the two proteins (stars) bars = 60 μm.

Confocal analysis of the patient’s skin revealed abnormal expression of K10 with respect to control skin, reflecting its achantolitic state (Fig 3A and 3B). The basal cell layer, highlighted by positivity for the basal marker p63, was expanded (Fig 3C and 3D). As a consequence, K14 expression persisted in the upper layers (Fig 3I–3L) and exhibited a modest overlap with K10 expression. At higher magnifications, K1/K10 tonofilaments were clearly visible, forming shells in the cytoplasmic peripheral area of the nucleus (Fig 3F and 3G). In addition, we observed the presence of several binuclear keratinocytes in the granular layer (Fig 3G, stars) and increased loricrin expression that was also abnormally present in the cell nucleus [6] (Fig 3H).

The presence of these clinical and ultrastructural findings, especially the presence of binucleated cells and EPPK, were consistent with the diagnosis of IH-CM.

### Genetic analysis

To confirm the diagnosis, we decided to perform a molecular investigation using the patient genomic DNA. All of the *KRT1* exons were PCR amplified and sequenced [6, 7, 17, 18]. However, no mutations were found in the region encoding the terminal V2 domain or the other domains of K1.

**Fig 4:**
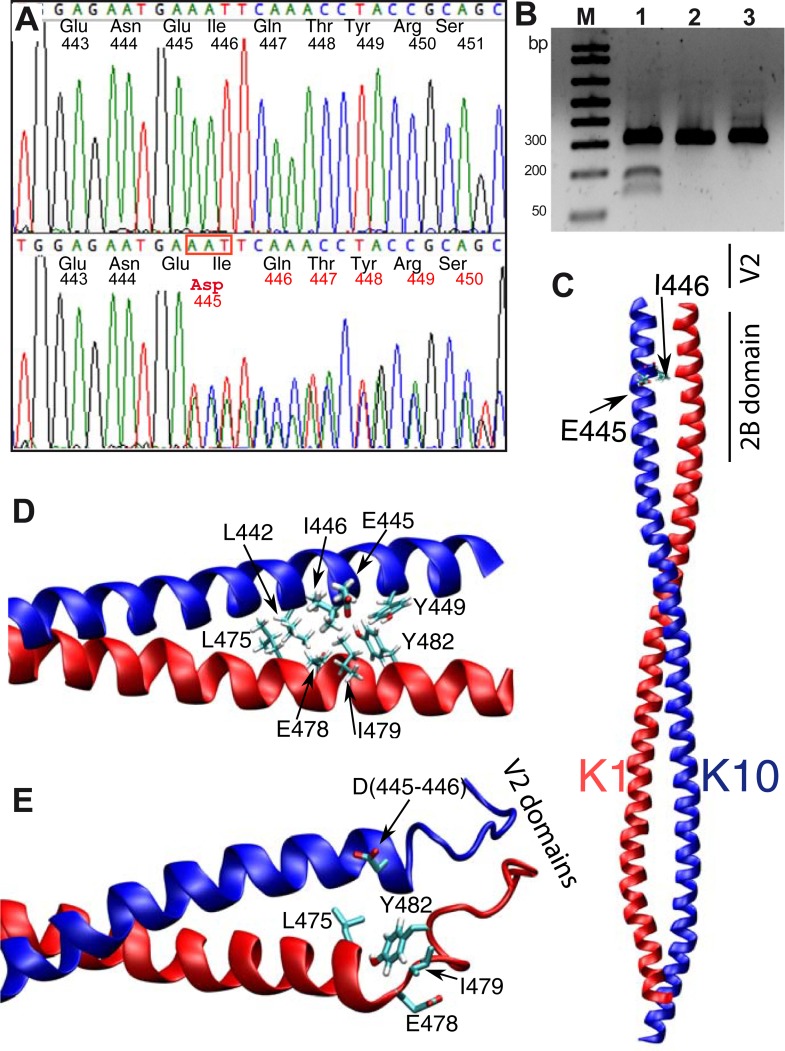
Genetic and structural results from *in silico* investigation. (A) Direct sequence of PCR amplified *KRT10* exon 6, showing the deletion, identified by a scrambled electropherogram from the patient (lower Panel) and the father’s samples (upper panel). (B). Bcl1 digestion analysis of the three family members, showing the presence of digested DNA only in the patient sample lane (lane 1). (C) 3D structure of the K1-K10 crystal structure (PDB id 4ZRY). Lateral chains of residues E445 and I446 in K10 are shown in licorice representation. (D) Close up view of the K1-K10 crystal in the proximity of the mutation. Inter-chain interactions are formed between L442, E445, I446 and Y449 in K10 with L475, E478, I479 and Y482 in K1 (lateral chains in licorice representation; mutated residues are highlighted with a larger bond dimension). (E) Snapshot of the dimer structure after 80 ns of Molecular Dynamics simulation. The rotation of the C-terminal region of the K10 helix is visible. Helix structures in the two proteins are conserved in the N-terminal region (Q447 in K10 and E478 in K19, while the respective C-terminal regions are highly disturbed.

Based on the fact that keratin proteins are expressed as heterodimers, and the companion of K1 in the suprabasal layer is K10, we searched for mutations in the coding region of the latter gene, despite the fact that generally the presence of palmoplantar keratoderma indicates K1 involvement. Remarkably, *KRT10* gene analysis identified the presence of the variation c.1334_1336delAAT in exon 6, which was also confirmed in the mRNA transcript. The mutation involves a deletion of three nucleotides, the last base of codon 445 (GAA), and the first two bases of codon 446 (ATT), thus generating a substitution of both p.Glu445 and p.Ile446 by pAsp (p.Glu445_Ile446delinsAsp), codified by the new codon GAT (Fig 4A); no other amino acid is affected, since the sequence reverts to frame. Since the mutation introduces the presence of a new Bcl I restriction site (TGAAATTCA-> T’GATCA), we used a restriction analysis to investigate the parental genome. As a result, Bcl I digestion of amplified exon 6 demonstrated that *KRT10* c.1334-1336delAAT is a “de novo” variation, since it is not present in the patient’s parents. These results were also confirmed by Sanger direct sequencing (Fig 4A). Digestion analysis was used to check fifty unrelated healthy people (100 alleles), in which the new mutation was not found. This strongly suggests that the mutation, which is not reported in the dbSNP databases, is responsible for the clinical phenotype.

### Model simulation

As shown in the structural model, the amino acid change is located next to the last heptad of the K10 2B domain (Fig 4C). According to the model, based on the previously published crystal structure [27], the inter-chain interactions are formed between L442, E445, I446 and Y449 of K10 with L475, E478, I479 and Y482 of K1 (shown in Fig 4D where the mutated residues are highlighted with a larger bond dimension). A simulation of the mutant structure obtained by eliminating the original E446-I456 residues and reconsidering the interactions with K1 residues under the presence of the mutant aspartic acid (most probable rotamer) was performed. The starting model was refined by geometry optimisation followed by molecular dynamic simulations with Gromacs (see materials & methods). The simulation (Fig 4E, S1 Movie) shows an alteration of the C-terminal portion of the alpha helical domain of both proteins. The substitution of residues 445–446 with an Asp produces the rotation of the C-terminal region of the K10 helix, due to the unfavourable interactions between the mutated residue and the interacting residues in K1. These preliminary results show that the helical structures in the N-terminal region of Q447 in K10, and E478 in K1, are conserved, while the respective C-terminal regions are highly perturbed (Fig 4E, arrow).

These data suggest a fundamental role for the correct 2B-V2 hetero-domain structure in the organisation of keratin intermediate filaments, thus linking the disease to the disruption of this structure.

## Discussion

The histological alterations of IH-CM involve the system of keratin filaments [3], which, starting from the first layer of suprabasal cells, group together, forming "shell-like" lengthened aggregates in the cytoplasm and around the nucleus. These features distinguish IH-CM from BCIE, and, clinically, they explain why vesicle-bullae are not manifested in a macroscopically visible manner. The clinical phenotype of our patient showed an intense and widespread hyperkeratosis and papillomatous appearance, excluding part of the face but including the palmoplantar areas. Blisters were absent, a feature supported by the absence of acantholysis in the histological evaluation, as well as by the presence of filament abnormalities and binucleated cells characteristic of this group of ichthyosis.

Currently has been proposed a classification of IH into two different forms, the Curt Macklin and the Lambert Type, on the base of palmoplantar skin involvement [25, 28]. According this hypothesis, the IC-CM is associated to *KRT1*, and the IH-L possibly associated to *KRT10* mutations, resembling the genotype phenotype association in BCIE, EHK, CIEH [29, 30] respect to the palm and sole involvement. This hypothesis is not consistent with our findings.

Our results demonstrate that IH-CM is a clinical entity caused by mutations in *KRT10* or *KRT1*, that modify the three-dimensional structure of the C-terminal domain formed by this specific keratin dimer. In fact, the shift of one amino acid due to the deletion mutation in the K10 2B domain causes a rotational change. This change disturbs the pairing of all subsequent amino acids, leading to the impairment of the correct interaction between the K1-V2/K10-V2 domains and resembling the effects of the deletion mutations previously described in *KRT1* [6, 7]. Mutations in the amino acids in position 445 and 446 have already been detected in the K10 protein. In the first case, the p.Glu445Lys substitution leads to the classic phenotype of BCIE [31], whereas the mutation p.Ile446Thr results in the phenotype described as annular epidermolytic ichthyosis [32], resembling the clinical and histologic features of both BCIE and Ichthyosis Bullosa of Siemens (IBS).

Importantly, in both cases, the structure of the KRT1/10 V2 domain is not modified, and palmoplantar region of the patients is not involved.

To finally distinguish the biochemical basis of the different diseases, deeper investigations into the biophysical structure of V2 domain by Cryo-EM or X-ray, with clear analysis of the resulting protein-protein interactome, are required. It is evident that individual mutations may lead to distinct clinical phenotypes. In fact, while our data indicate that the patient conforms to the clinical and pathological criteria of ichthyosis hystrix Curth Macklin type, the detailed analysis reveal how complex this pathology can be.

Indeed, our data highly suggest a fundamental role of the KRT1/10 V2 domain in IH-CM pathogenesis, indicating that the clinical phenotype of a specific keratin disease is not only dependent on the affected keratin or on a specific amino acid change but also relies on changes in defined conformational domains [22, 27] that are responsible for specific functions.

## Conclusions

After the description of the “porcupine man” in 1954, other cases corresponding to the diagnostic criteria were published, and there is still not a clear and worldwide-accepted classification of Ichthyosis hystrix. The result of this current work now demonstrates that ichthyosis hystrix of Curt Macklin is associated to both *KRT1* and *KRT10* mutations, when they lead to the disruption of the correct 2B-V2 hetero-domain, linking the disease to the loss of function of this structure.

A potential relationship between the large K10 allele [33] and PPK phenotype has been suggested in a linkage analysis study involving a large Uzbeks family [34]. However, this study fails to find a mutation in *KRT10* gene due to a partial Sanger sequencing. Therefore, this work produces now the first evidence of a *KRT10* heterozygous dominant mutation associated with PPK.

## Supporting information

S1 Fig Heptad structure of the N-terminal rod domain. Schematic representation of the heptad (a-b-c-d-e-f-g) of the HTM of K10 (left) and K1 (right), showing hydrophobic interactions between positions “**a”** and “**d**” and ionic hydrogen interactions between positions “**e”** and “**g”**, that are abolished by the mutation.
(PDF)

S1 Movie Video showing a 16 second simulation of the interaction between K1 and K10 terminal domains. (MPG)

## Abbreviations

IH: Ichthyosis hystrix
IH-CM: ichthyosis hystrix Curth-Macklin
IH-L: Ichthyosis hystrix Lambert type
KIF: keratin intermediate filaments
PPK: palmoplantar keratoderma
