# Supplementary figures and images for: Role of the keratin 1 and keratin 10 tails in the pathogenesis of ichthyosis hystrix of Curth Macklin
Source: PLoS One. 2018 Apr 24;13(4):e0195792. doi: 10.1371/journal.pone.0195792 (PMC5918167; doi:10.1371/journal.pone.0195792)

Figure Supplementary 1

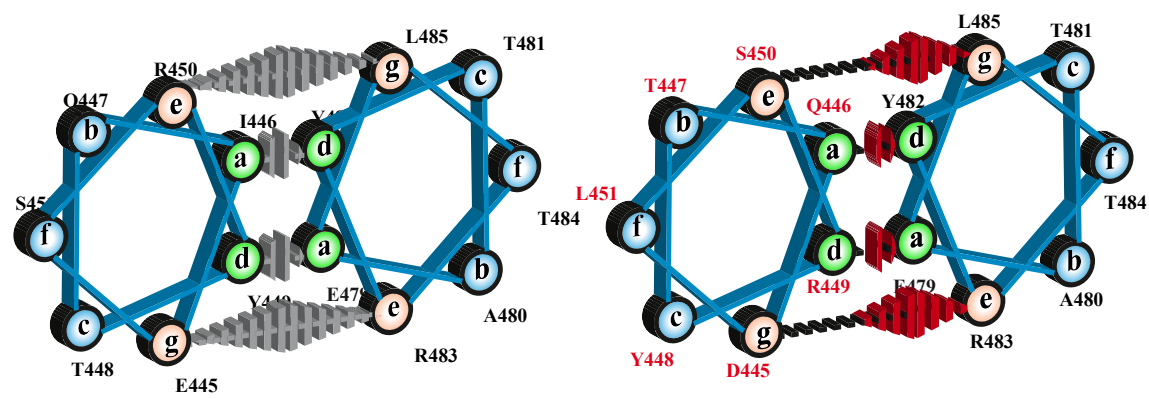

Supplement: S1 Fig — Schematic representation of the heptad (a-b-c-d-e-f-g) of the HTM of K10 (left) and K1 (right), showing hydrophobic interactions between positions “a” and “d” and ionic hydrogen interactions between positions “e” and “g”, that are abolished by the mutation. (PDF) [file pone.0195792.s001.pdf]
